# John Todd (1914–1987)

**DOI:** 10.1007/s00415-024-12846-8

**Published:** 2024-12-16

**Authors:** Andrew J. Larner, Gashirai K. Mbizvo

**Affiliations:** 1 Department of Brain Repair & Rehabilitation, Institute of Neurology, University College London, London, UK; 2 Pharmacology and Therapeutics, Institute of Systems, Molecular and Integrative Biology, University of Liverpool, Liverpool, UK; 3 Liverpool Centre for Cardiovascular Science, University of Liverpool and Liverpool Heart & Chest Hospital, Liverpool, UK

John Todd was a clinician whose training and practice were those of a psychiatrist, so it may seem paradoxical to claim him as a “Pioneer of Neurology”. Nevertheless, his astute clinical observations on perceptual and delusional disorders have impacted neurological diagnosis and thinking.

Born in London, Todd trained at King’s College Hospital, London, qualifying in 1938. During the Second World War, he served in the Royal Army Medical Corps before undertaking his training in psychiatry. This included four years at Littlemore Hospital in Oxford, a location of possible relevance to his most noted contribution to neurological nomenclature. In 1955, Todd was appointed Consultant Psychiatrist to High Royds Hospital in Menston, near Leeds in West Yorkshire, sometimes known as Menston Hospital, but initially called the third West Riding of Yorkshire County Asylum when it opened in 1888. He remained at Menston until his retirement in 1979 [1].

Todd’s principal claim to neurological attention rests on his characterisation of perceptual distortions, both visual (metamorphopsia) and somaesthetic (micro- and macrosomatognosia), as the “syndrome of Alice in Wonderland” [2], named after the eponymous heroine of the story by Lewis Carroll, pseudonym of the Oxford mathematician Charles Lutwidge Dodgson (1832–1898). Todd’s choice of this terminology was based on the resemblance of the bizarre subjective symptoms described by six patients referred to psychiatric clinics with the graphic illustrations of Alice’s predicament as pictured by Sir John Tenniel in the original 1865 publication of Carroll’s book. These symptoms also included seeing objects as more distant (teleopsia) or close (peliopsia) than they actually were, and duplication (complete, or partial, e.g. of the head) or absence of body parts (e.g. an arm). Most occurred in the context of a current or past history of migraine. Although, as Todd acknowledged, some of these clinical phenomena had been previously described in the context of migraine [3], the “Alice in Wonderland syndrome” nomenclature helped to raise awareness of symptoms which patients were often afraid to speak about for fear of being considered insane. Of note, Todd ascribed these perceptual symptoms to faulty integration of the visual, kinaesthetic, tactile, auditory, and psychical components making up the body image. Moreover, he suggested these symptoms had localising value, to a site of origin in the parietal lobe, but he did not identify their lateralising (generally right-sided) value. Alice in Wonderland syndrome remains of interest to neurologists as one of the “pathologies of sensory input” [4].

Todd also published on other perceptual disorders, including autoscopia and drug-induced Lilliputian hallucinations (micropsia). Duality was also a subject of interest, including “shadow doubles” and their projection as an hallucinatory Doppelgänger. He was also interested in Capgras syndrome, the delusion of doubles, the misidentification of a familiar individual as an imposter [5].

**Fig. 1:**
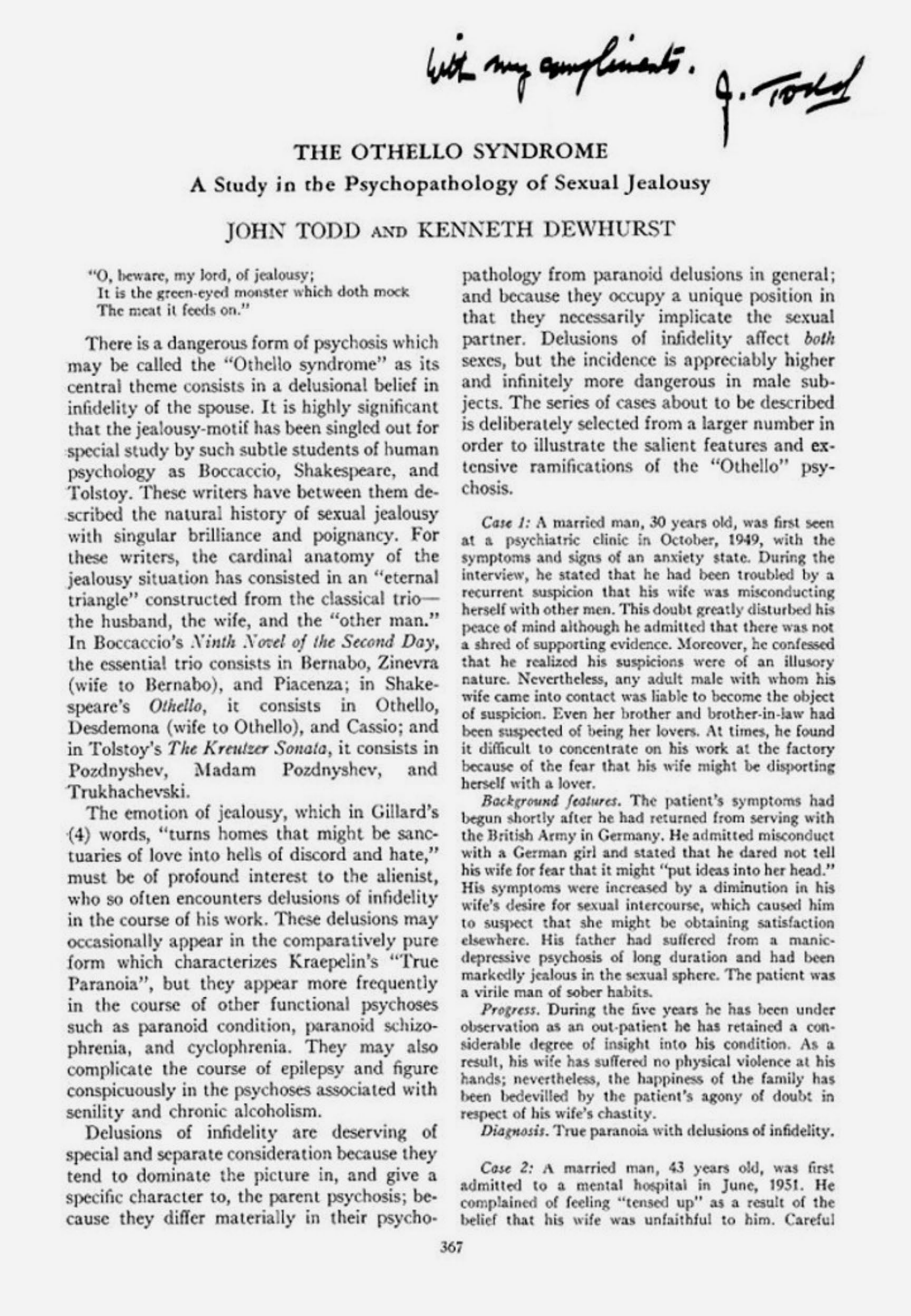
Title page of Todd and Dewhurst’s 1955 paper on the Othello syndrome [6] with Todd’s signature borrowed from another paper

Todd’s reference to a literary character was once again instrumental in drawing attention to a previously described neuropsychiatric disorder, namely the particular delusion of pathological or morbid jealousy, often associated with preoccupations of infidelity in the absence of legitimate proof. He christened this the “Othello syndrome” (Fig. 1) [6] after the titular character of Shakespeare’s play, but also referred to similar accounts in the literary works of “subtle students of human psychology” such as Bocaccio and Tolstoy (*The Kreutzer Sonata*), as well as Robert Burton’s *The Anatomy of Melancholy*. He later drew attention to hypophallism as a real or imaginary cause of morbid sexual jealousy [7]. Although initially described in association with primary psychiatric disorders, Todd reported that Othello syndrome might also complicate epilepsy and occur in the psychoses associated with “senility” and chronic alcoholism [6]. Othello syndrome may in fact be more common in the context of neurological disorders, predominantly neurodegenerative disorders including Parkinson’s disease, and also with structural lesions, especially of the right frontal lobe.

The publication of Todd’s papers on “Alice in Wonderland syndrome” and “Othello syndrome” in journals based in North America may have heightened awareness of his work, likewise the inclusion of “Othello syndrome” in Enoch and Trethowan’s book on *Uncommon Psychiatric Syndromes* first published in 1967. Certainly, Todd’s proposed nomenclature for both syndromes was adopted relatively rapidly.

Several of Todd’s papers were co-authored with the psychiatrist and medical historian Kenneth Dewhurst (1919–1984), one of whose interests was John Hughlings Jackson’s (1835–1911) contributions to psychiatry [8]. Todd’s characterisation of the Capgras syndrome was essentially Jacksonian: “the disintegration of the personality doubtless sets free primitive modes of thoughts, which include the tendency to think in terms of doubles and dualisms” [5].

Todd also had an interest in medical history, to which he made a number of contributions, some pertinent to neurology. His very first paper, from 1954, was an account of the physician and author Anton Chekhov (1860–1904) and he later wrote on the Brontë children. However, in retirement his focus turned to the first West Riding of Yorkshire County Asylum, opened at Wakefield in 1818, and by this time called Stanley Royd Hospital. In particular, Todd’s research was on the period in the late 1860s and early 1870s when the Wakefield Asylum was under the superintendency of James Crichton-Browne (1840–1938) and associated with the work of two neurological pioneers, David Ferrier (1843–1928) and Hughlings Jackson. It was at Wakefield that Ferrier’s initial experimental studies in cortical localisation, to test the clinical inferences of Hughlings Jackson, took place. Both published in the house journal, the *West Riding Lunatic Asylum Medical Reports*. Ferrier’s results had a major impact on the practice of clinical neurology, confirming it as a discipline underpinned by localisation, an approach which also paved the way for the development of neurosurgery. With the former secretary of Stanley Royd Hospital, Lawrence Ashworth, who had ensured the survival of the old Asylum records and helped to establish a museum at the hospital, Todd authored two substantial historical pieces on the Asylum, but sadly neither was published before his death [9, 10]. Beyond their local interest, these works continue to inform scholarship on asylum history generally and also serve to illuminate a key period in the history of neurology. (For a bibliography of Todd’s papers, see www.highroydshospital.com/galleries/the-career-of-dr-john-todd-and-drug-addiction-case-studies/).
